# Pilot deployment of a cloud-based universal medical image repository in a large public health system: A protocol study

**DOI:** 10.1371/journal.pone.0307022

**Published:** 2024-08-29

**Authors:** Viviane Margarida Gomes Pacheco, Joselisa Peres Queiroz Paiva, Brunna Carolinne Rocha Silva Furriel, Paulo Victor Santos, José Raniery Ferreira Junior, Marcio Rodrigues Cunha Reis, Daniel Tornieri, Guilherme Alberto Sousa Ribeiro, Luan Oliveira Silva, Solange Amorim Nogueira, Rafael Maffei Loureiro, Wesley Pacheco Calixto

**Affiliations:** 1 Hospital Israelita Albert Einstein, Sao Paulo, Brazil; 2 Electrical, Mechanical & Computer Engineering School, Federal University of Goias, Goiania, Brazil; 3 Technology Research and Development Center (GCITE), Federal Institute of Goias, Goiania, Goias, Brazil; Alma Mater Studiorum Universita di Bologna: Universita degli Studi di Bologna, ITALY

## Abstract

This paper outlines the protocol for the deployment of a cloud-based universal medical image repository system. The proposal aims not only at the deployment but also at the automatic expansion of the platform, incorporating Artificial Intelligence (AI) for the analysis of medical image examinations. The methodology encompasses efficient data management through a universal database, along with the deployment of various AI models designed to assist in diagnostic decision-making. By presenting this protocol, the goal is to overcome technical challenges and issues that impact all phases of the workflow, from data management to the deployment of AI models in the healthcare sector. These challenges include ethical considerations, compliance with legal regulations, establishing user trust, and ensuring data security. The system has been deployed, with a tested and validated proof of concept, possessing the capability to receive thousands of images daily and to sustain the ongoing deployment of new AI models to expedite the analysis process in medical image exams.

## Introduction

Nowadays, multi-expert clinical studies often span months or even years, accumulating image data from different institutions. This presents a significant challenge in efficiently managing such vast data, which is pivotal in large-scale clinical image studies and research. These studies are often subject to strict regulatory requirements, including patient privacy protection [1]. The dynamic field of efficient data management focuses on strategies to select and filter context-specific information, aiming to alleviate the burden of data overload for processing and storage. Within this domain, methods for organizing and indexing data are being developed to improve access and retrieval. This effort marks an ongoing dedication to refining data management practices across various application areas [2, 3].

In the modern era, effective data management is critical for large-scale studies and patient care [1]. The medical community widely recognizes the importance of data sharing to expedite progress, minimize costs, and facilitate reproducible research. [4]. In clinical settings, the selection, organization, and proper availability of data depend on efficient storage systems that are scalable, high-performing, and reliable. Distributed storage systems, such as MongoDB [5] and Hadoop Distributed File System (HDFS) [6], are notable for managing large data volumes. Additionally, cloud-based storage solutions such as Amazon Simple Storage Service (Amazon S3) [7] and Google Cloud Storage [8] are increasingly used for image archiving and communication, offering the necessary scalability and durability. Efficient data management not only focuses on storage but also on developing strategies to manage the growing amount of data, ensuring its effective use across various contexts [1].

Current systems, while effective for individual clinical workflows, encounter difficulties in shared workflows, particularly in integrating with third-party applications for data handling and analysis [1]. This field is continuously evolving, marked by significant contributions such as those of Greenes *et al.* [9]. Successful data management deployment demands a thorough evaluation of technologies, system architecture, and the specific context of data usage. Each application domain might have distinct requirements for data structuring, processing, storage, and retrieval [10]. Despite increased data accessibility, managing and analyzing it at scale remains a challenge, as existing systems are typically designed for linear workflows. Anticipating data volume growth is necessary, requiring an analysis of the system’s storage and processing capacity over time. The selected technologies must be capable of scaling with data volume increases while ensuring data availability and integrity [11].

Currently, data management encompasses a range of technologies, including relational databases, NoSQL, cloud storage, distributed processing platforms, and streaming technologies. The selection of these technologies hinges on specific requirements, considering scalability, performance, security, and the potential for Artificial Intelligence (AI) applications. In AI, it is necessary to address the entire workflow for efficiency. Integrating AI models into production systems poses technical challenges and broader issues such as ethics, regulations, user trust, and security [12]. Examples of efficiency in this domain include the KeystoneML system, demonstrating scalable training in distributed environments [13], and Google’s TensorFlow Extended (TFX) platform, offering a comprehensive solution for learning tasks and continuous training with user-friendly, configurable tools that ensure reliability and scalability in production settings [14].

Numerous studies have focused on automating tasks and leveraging technologies for information management in healthcare, a sector characterized by a large volume of digital data. The World Health Organization highlights digital health’s role in enhancing data collection, analysis, and informed decision-making [15]. In Brazil, the Unified Health System (SUS) deals with a vast amount of data due to the country’s diverse demographics, including patient records, diagnoses, treatments, surgical procedures, laboratory tests, and epidemiological data [16]. The SUS manages this information to monitor population health, formulate public policies, and evaluate medical interventions, thus aiding in informed decision-making. The digitization and integration of healthcare systems have created an extensive data repository, providing valuable insights for healthcare professionals, researchers, and policymakers, aimed at improving care quality and public health.

From 2005 and 2008, the SUS conducted 166,514 procedures in 180 hospitals, with an average mortality rate of 2.33%, varying regionally. By 2015, the SUS served 71.5% of Brazil’s population [17, 18]. In particular, approximately 80% of the percutaneous coronary interventions in Brazil are performed by SUS, in a country with around 203 million people according to the 2022 Census. SUS’s core principles ensure universal healthcare access, irrespective of individuals’ socio-economic, educational, or occupational backgrounds. Although it has evolved since Brazil’s 1988 Constitution, the SUS continues to need development, especially with changing health needs and disease prevalence [19]. A central aim of SUS is to maintain excellent medical care, considering each individual’s biopsychosocial context [20]. However, the SUS faces challenges, including inconsistent protocols for diagnostic imaging [21, 22], regional shortages of specialized doctors [18], and inadequate infrastructure for sustainable data storage and long-term access [23].

To address the deficiency in sustainable, long-term storage and access to healthcare data, the Brazilian government, via the Ministry of Health, launched a project to create a cloud-based, universal database platform PI. This platform aims to standardize access to high-quality medical exams nationwide. A key innovation of this project is the consolidation of a patient’s exams in one location, simplifying access for multi-disciplinary specialists. This integration is enhanced by AI-driven diagnostic tools, fostering collaboration among researchers, companies, and stakeholders, thus supporting the SUS. Advanced computer system technologies are employed to implement this project, focusing on developing a universal platform that will store and manage various imaging exams conducted by SUS, enabling access via electronic devices and applying AI-based technologies to assist in diagnostic decisions.

## Technological infrastructure, innovations, and tools in the healthcare context

In the deployment of the universal platform, the Picture Archiving and Communication System (PACS) is employed, a healthcare technology dedicated to the storage, management, and provision of medical images, such as radiographs, computed tomography (CT) scans, and magnetic resonance imaging (MRI) [24]. PACS has replaced conventional radiographic films with digital images, enabling quick and secure access to this data by healthcare professionals. Additionally, it facilitates communication across different departments and specialties, enhancing efficiency in patient diagnosis and treatment [25]. Medical images are typically stored in the Digital Imaging and Communications in Medicine (DICOM) format, a globally recognized standard in healthcare communication. Despite its widespread use [26], variations in DICOM syntax across different PACS vendors and imaging devices can create challenges in data integration.

To address these issues, the Vendor Neutral Archive (VNA) steps in. It resolves systems incompatibilities by deconstructing PACS data before transferring it to a new repository. This ensures accurate syntax interpretation [27]. Acting as a bridge between different image formats and PACS systems, the VNA uses algorithms to query and standardize data, aligning it with the DICOM standard [28]. Additionally, the VNA interfaces with electronic records, adhering to the HL7 standard. As a vendor-independent intermediary, it significantly enhances the efficiency of medical information management [29, 30]. For optimal functionality, the VNA must feature a multi-location architecture to guarantee interoperability and access to images and clinical data [31, 32].

The VNA streamlines healthcare data management by consolidating images from multiple sources, enabling visualization through third-party applications, and enhancing information exchange [30]. To safeguard privacy, the VNA implements robust measures including security protocols, encryption, user authentication, and adherence to HIPAA standards [33, 34]. A key element is the anonymizer, which protects patient confidentiality by replacing personal details in medical records with codes or fictitious data [35]. This facilitates the use of these records for research, training, and analysis without compromising patient identity, thereby bolstering the security and integrity of the VNA and aiding in healthcare advancements [36].

For the successful deployment of a universalized platform, it is critical to follow protocols such as the ISO 8000 series, initially approved in 2009 and continuously updated since 2017. Established by the International Organization for Standardization, these standards provide guidelines for data management and quality improvement [37]. The purpose of these standards is to assist organizations in improving the accuracy and reliability of data in their processes and operations. They cover fundamental concepts to specific requirements for exchanging messages containing computer-transmitted data. The ISO standards, including ISO 8000-1, ISO 8000-110, ISO 8000-150, ISO 8000-2, ISO 8000-51, and ISO 8000-62, outline concepts, requirements for data exchange, functions and responsibilities for data quality management, terms and vocabularies, exchange of data policy statements, and methods for assessing the maturity of organizational processes, respectively [38–43]. They are designed to enhance the effectiveness, efficiency, and reliability of data-dependent operations across different sectors [44].

Comprehensive protocols for data management system deployment encompass guidelines for technology infrastructure, deployment, security, monitoring, documentation, training, and data protection [45, 46]. Adhering to these protocols ensures system uniformity and quality, reduces risks, and enhances data governance [47]. In the context of data protection, the General Data Protection Regulation (GDPR) in the European Union, deployed in 2018, establishes comprehensive rules for protecting personal data, promoting responsibility, mandating breach notifications, and regulating international data transfers [48]. In Brazil, the General Data Protection Law (LGPD) shares similar principles for protecting of personal data [49].

In 2022, the American Data Privacy and Protection Act (ADPPA) undergoes approval with amendments, having been validated by the House Energy and Commerce Committee and awaiting approval in the U.S. House of Representatives [50]. This development is significant as the United States, unlike the European Union, does not have a comprehensive federal data privacy law and primarily relies on state laws and sector-specific regulations in fields like health, finance, and telecommunications [51]. Within this framework, ISO 27701 stands out, outlining requirements for Privacy Information Management Systems (PIMS) and offering guidance for personal data controllers and processors [52]. In data management system deployment, standards such as the Capability Maturity Model Integration (CMMI) and Brazilian Software Process Improvement Reference Model (MPS.Br) are important. These standards optimize software development and project management [53, 54]. In Europe, ISO/IEC 15504, also known as Software Process Improvement and Capability Determination (SPICE), provides a globally recognized framework for assessing and improving software processes [55].

This research emphasizes the critical need for efficient and secure management of medical images within large healthcare systems. The categorical hypothesis is that integrating PACS with VNA, coupled with AI models, can result in a diagnostic tool to enhance medical assessments and aid in disease detection. The primary objective is to create a universal platform for storing medical images, ensuring nationwide access. This objective involves establishing a universal cloud-based database, developing digitization and integration solutions for exams, automating diagnostics, and integrating AI in medical diagnosis. These advancements aim to enable precise monitoring of pathologies, though they require significant investment. The relevance of these improvements extends globally, impacting healthcare institutions of all sizes, including those within Brazil’s SUS.

## Methodology

**Fig 1:**
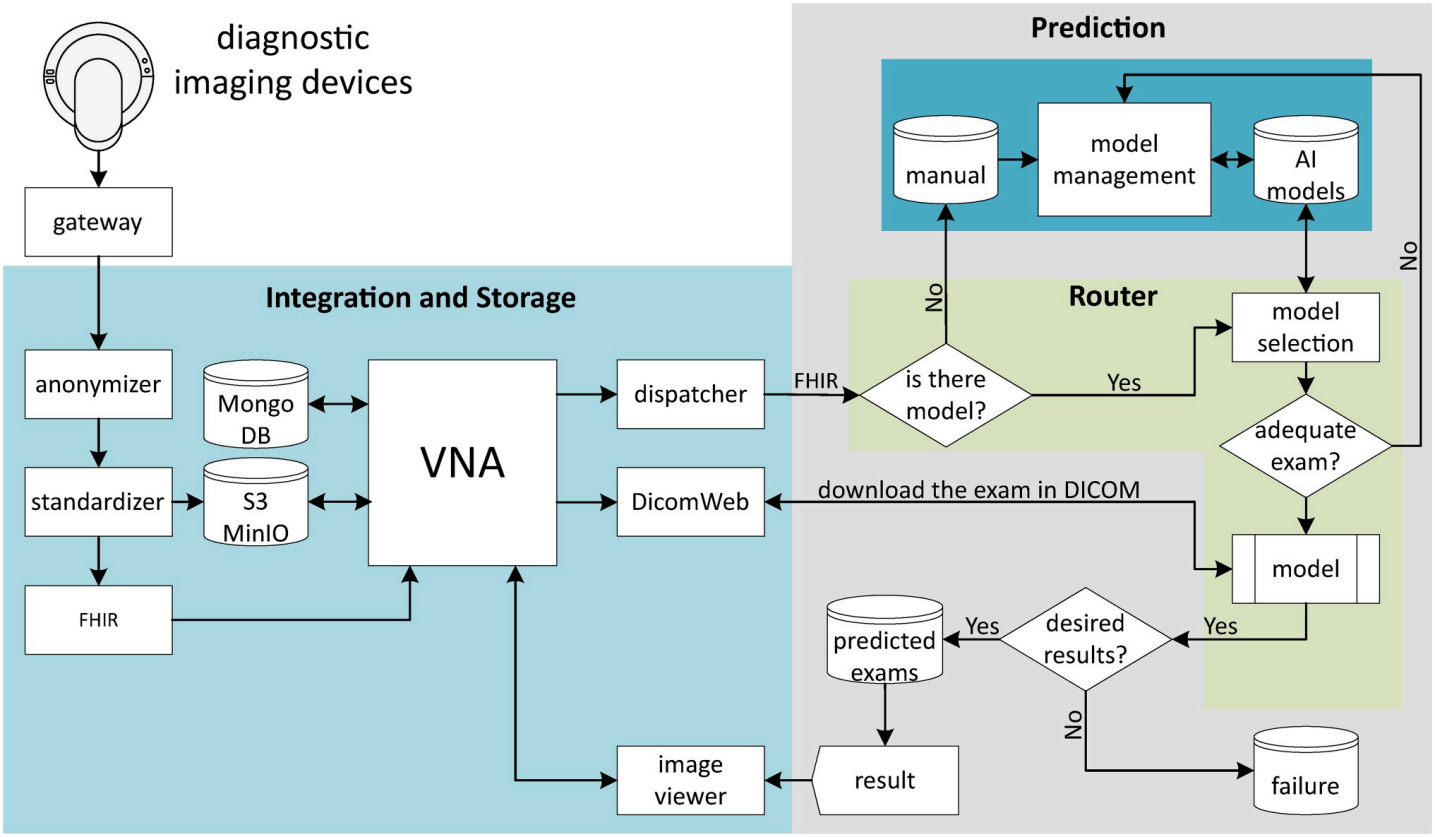
Flowchart of the proposed methodology.

This study adopts a hypothetico-deductive research approach [56], focusing on the practical deployment of a medical image storage platform. The aim is to provide healthcare professionals with consistent, high-quality access to exam data, irrespective of their geographical location or the timing of access. The workflow starts with conducting exams using diagnostic imaging devices and culminates with medical experts comparing these original exams against analyses produced by AI models. Fig 1 depicts the flow of this proposed methodology.

### Collection, storage, and integration

**Fig 2:**
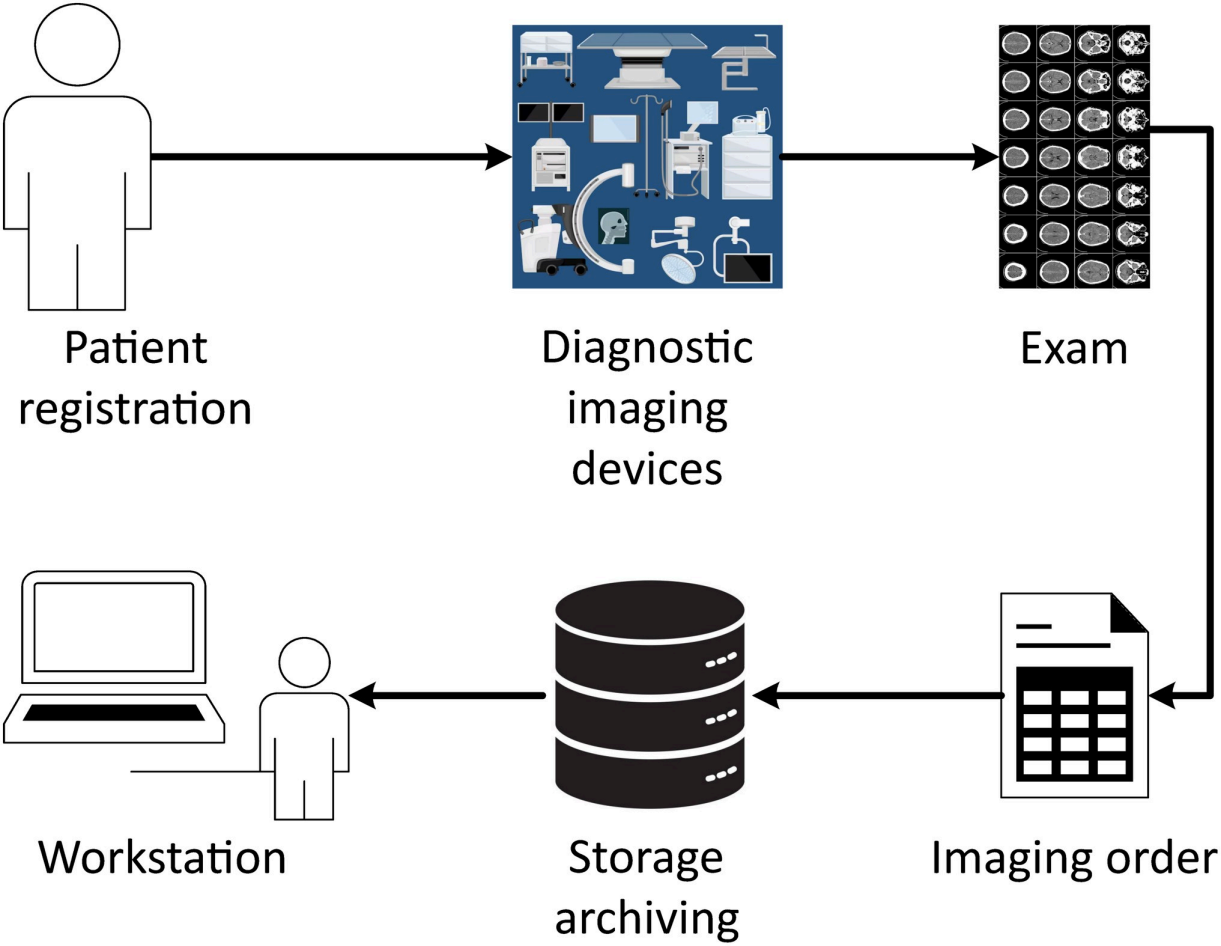
Data collection cycle by a specific department or institution.

The data collection process initiates with the patient providing their information upon arrival at the examination area. Following registration and identification of the examination type, the patient proceeds to the imaging diagnostic device for the procedure. However, not all imaging devices automatically deliver images in the DICOM format. The use of this standard varies depending on the device type, manufacturer, and the healthcare setting’s specific needs. Certain older devices or those used in specialties like endoscopy or ophthalmology often produce images in proprietary formats, necessitating their conversion or export of the images and metadata to DICOM. Fig 2 displays the typical operational cycle in imaging diagnostic settings, highlighting the handling of various examination formats, both DICOM and proprietary. After storage, these examinations are accessible for further evaluation and annotation by specialist doctors.

Diagnostic imaging protocols vary across different modalities and clinical areas, yet share key common elements. These elements include pre-examination preparation instructions, patient positioning guidelines for optimal image acquisition, and technical parameters such as radiation exposure and scanning duration. In MRI and CT scans, protocols are tailored with specific sequences for various body parts or clinical conditions. When contrast agents are used, guidelines cover their application, necessary precautions, and patient monitoring. Maintaining a detailed record of patient information, clinical conditions, and procedural specifics is necessary. Quality criteria ensure image suitability, while post-examination care guidelines are also provided. These generic elements are adaptable to each modality and clinical area’s unique requirements. Standardizing these protocols enhances the quality and reliability of diagnostic imaging results.

The proposed methodology begins after examinations are complete. As illustrated in Fig 2, examinations first pass through a **gateway** before reaching the PACS. This **gateway** ensures the secure transfer of DICOM images and other formats between institutions. In the PACS, illustrated in Fig 1, the **anonymizer** processes the examinations to protect patient confidentiality by removing identifiable information. This de-identification is done using the Clinical Trials Processor (CTP) DICOM Pixel Anonymizer (DICOM Pixel Anonymizer: https://github.com/susom/mirc-ctp) [57]. The DICOM Pixel Anonymizer uses a script to decide which parts of the image to erase, based on information not contained in the DICOM file pixels. This step involves analyzing incoming DICOM objects to inspect if they are images, and if so, checking their compression status. When images meet the specified criteria, the **anonymizer** executes the script to remove selected areas. These areas are precisely defined by rectangular coordinates. If an object does not meet the criteria, it remains unmodified and passes through the system.

Following de-identification, the examinations proceed to the **standardizer**, as illustrated in Fig 1. This tool corrects specific fields in the DICOM images, aiding **AI models** in recognizing the type of examination and the body areas examined. After standardization, the data is transferred to AWS S3/MinIO, which constitutes the set of storage systems with an abstraction layer. This abstraction layer, termed ‘abstrador’, decouples the object storage system from the rest of the project. The abstraction layer’s role is to provide a consistent interface that encapsulates the complexities of the underlying storage provider. This allows other project components to interact with the abstraction layer without needing to deal with the specific nuances of each storage provider. This design makes the project vendor-agnostic, avoiding reliance on any specific provider and enabling a seamless transition between different storage services (cloud or on-premise infrastructures). Following the **standardizer** and ensuring that all examinations are in DICOM format, the Fast Healthcare Interoperability Resources (FHIR) [58] protocol is employed. Developed by HL7, the FHIR standard streamlines the exchange of electronic health information, enhancing the integration of healthcare systems and standardizing data sharing.

With the integrated system in place, all data is forwarded to the VNA. The VNA is connected to AWS S3/MinIO, an encrypted database that maintains correspondences between the original examination dataset and the anonymized examination dataset. This process is utilized to link the original examinations with the examinations that will undergo computational manipulations, being necessary for data integration, migration, and transformation, ensuring consistency and correspondence between the examinations. Thus, during computational manipulation, the patient’s identity associated with the examination under analysis is kept confidential. However, after the entire process, the specialist doctor needs to compare the original examination with the analyses generated by the **AI models**. The technique used for creating the encrypted identification pointer is developed in the Research Electronic Data Capture (REDCap) Web Platform for data collection and management [59, 60]. In REDCap, access numbers identifying the anonymized examinations are registered, establishing the connection with the examinations stored in AWS S3/MinIO. Only the specialist doctor associated with the patient has permission to access the identification number in REDCap.

The VNA also connects to the NoSQL/MongoDB database, used for flexible and scalable data storage, employing JSON or BSON documents in collections. NoSQL/MongoDB is chosen for its ability to handle semi-structured data, horizontal scalability, and support for complex operations, proving especially useful in environments with rapid development requirements, efficient storage of large data volumes, and dynamic data. At the output of the VNA, there are the **dispatcher** and the DICOMWeb. The **dispatcher** notifies the Integration and Storage System to send a new examination, providing a delayed notice to ensure that all information from the previous examination has arrived in the Prediction System.

The **dispatcher** connects directly to the **router**, which confirms the arrival of all examination information. The **router** analyzes the information contained in the forwarded examinations, identifying the type of examination and verifying whether the AI model for that examination exists in the AI model database. Between the **dispatcher** and the **router**, the information is in FHIR standard, however, when manipulating data in the **AI models**, DICOM formats are required. Thus, when it is necessary to manipulate the examinations in the **AI models**, DICOMWeb is requested. DICOMWeb is the set of web services that uses the principles of Representational State Transfer (RESTful) architecture (These principles guide the design of Web distributed systems, prioritizing a unique representation of resources and stateless communication, promoting flexibility and interoperability in Web APIs). for communication and information exchange in DICOM-format images, adapted for web-based environments and promoting interoperability among different systems and providers.

### Deployment of a model to assist in diagnoses

If the **router** identifies that the model for the examination does not exist, it is sent to the **manual analysis** database. The information that the model is nonexistent is automatically forwarded to the **model management** system. If the AI model exists, the examination is directed toward an analysis of compatibility (**adequate exam**) parameters with the model. If the examination has all the necessary parameters, the AI model is applied to the examination. If the results of the manipulation by the AI model are the **desired results**, the examination is sent to the **predicted exams** database, otherwise, the examination is sent to the **failure** database.

In the case where the examination lacks all the necessary parameters, it is routed to the **model management** system. The **model management** system provides access to exam types that do not have defined models, to exam types that were not **adequate** with pre-existing models due to a lack of parameters, and to all registered models. In the **manual** database, examinations can be manually analyzed or wait for a model to analyze them. In the AI model database, all existing models for diagnostic analysis are stored, and these models can be in any language, as the platform is language-agnostic.

In the Prediction System, all logs of exams with unsatisfactory results are forwarded to the **failure** database for manual analysis. Exams with satisfactory results are sent to the **predicted exams** database, making them available for consumption by the **image viewer** of the VNA. The image viewing system is based on the Open Health Imaging Foundation (OHIF) [36, 61]. In this tool, it is possible to compare the original exam in the VNA with the predicted exam, presenting heatmaps, and segmentations, among other features. Only the physician responsible for the patient has access to the original exams and the exams predicted by **AI models**.

### Analysis, testing, and validation

The analysis, testing, and validation of the proposed system are fundamental processes to ensure successful deployment. Efficiency, security, and compliance with clinical and regulatory requirements are necessary to ensure safety and accuracy in examinations [62]. These stages include requirements analysis to understand the specific functionalities needed, detailed test planning identifying critical cases and acceptance criteria, and unit testing to verify the proper functioning of each component [63]. Integration tests ensure efficient communication between different modules of the proposed system, while performance tests assess behavior under different conditions, including maximum load [64].

Security is specifically tested to identify and address potential vulnerabilities in the system. Compliance tests are conducted to verify adherence to standards and regulations, such as the Health Insurance Portability and Accountability Act (HIPAA) (U.S. legislation from 1996 that protects health information, establishes privacy and security standards, and regulates the electronic transfer of data. Applicable to healthcare organizations and professionals, it imposes strict protocols and penalties for non-compliance). Disaster recovery capability is assessed to ensure operational continuity in adverse situations [63]. Additionally, clinical validation is performed through clinical tests involving healthcare professionals to ensure the system meets clinical needs and provides accurate and reliable results [65]. User training is provided to ensure a comprehensive understanding of the system and its functionalities [64].

Continuous evaluation is deployed to monitor the system’s performance and make updates as necessary [62]. All these tests are thoroughly analyzed and validated to ensure the system is robust, secure, efficient, and complies with clinical and regulatory requirements. Collaboration among Information Technology (IT) teams, healthcare professionals, and system providers is necessary to achieve maximum efficiency in system deployment [63]. Although the initial deployment cost is high, uniform and defined criteria are necessary for cost calculation, leading to savings over time after deployment [24]. In 1992, Choplin *et al* [66] emphasized that the goals of such a system are to improve operational efficiency while simultaneously maintaining or enhancing diagnostic capacity [66].

## Establishing protocol viability

Although the protocol study is a detailed plan that outlines the objectives, design, methodology, and organization of the research, some results were included to reinforce the feasibility of the proposal. The Integrated System for Medical Image Repository (ISMIR) is currently in the deployment phase following the successful completion of the proof of concept, which has been tested and validated. In response to a request from the Ministry of Health of Brazil, AI models have been developed and deployed for tuberculosis, melanoma, and Zika virus [67–72]. Currently, AI models for pulmonary embolism classification, cranial segmentation, and left ventricle wall identification are in the testing phase, presenting three different AI models for diagnostic solutions. All these cases have had their data inserted into ISMIR, and from the entire workflow illustrated in Fig 1, the models were implemented, tested, and validated. These three case studies are fully detailed in the works of Nogueira *et al.* [73], Santos *et al.* [74] e Silva *et al.* [75].

### Identifying ventricular walls in myocardial scintigraphy

In this study, the proposal is to use AI techniques, specifically the nnU-Net convolutional neural network, to enhance the identification of left ventricular walls in myocardial perfusion scintigraphy (MPS) images, aiming to improve the diagnosis and treatment of coronary artery disease. The methodology included data collection in a clinical setting, followed by data preparation and analysis using the 3D Slicer platform for manual segmentation and subsequently the application of AI models for automated segmentation. A total of 83 clinical routine exams were collected, each containing 50 slices, totaling 4,150 images, at the Albert Einstein Hospital, São Paulo, Brazil, during February and March 2023. For the MPS procedure, the standard protocol with acquisition in the supine position at rest was employed. The inclusion criterion was the signing of the Informed Consent Form (ICF), including all patients who underwent the examination during the collection period and signed the ICF. The exclusion criterion was the non-signing of the ICF.

All exams had reports, but only copies of the DICOM images were used for this study, with the originals stored in the PACS. Demographic data were collected and recorded in REDCap by the principal investigator, with no other researchers having access to sensitive patient data. After collection, the exams were anonymized to ensure patient privacy during analysis. Only the principal investigator has access to the *I*_*N*_ associated with the exam, stored in the REDCap form. A total of 83 patients were analyzed, aged 25 to 88 years. Of the 83 exams, 43 were used for validation, and three were randomly selected for submission to specialists. Each exam contains 50 slices, totaling 150 images per specialist. The remaining exams were divided into 31 for training and nine for testing.

**Fig 3:**
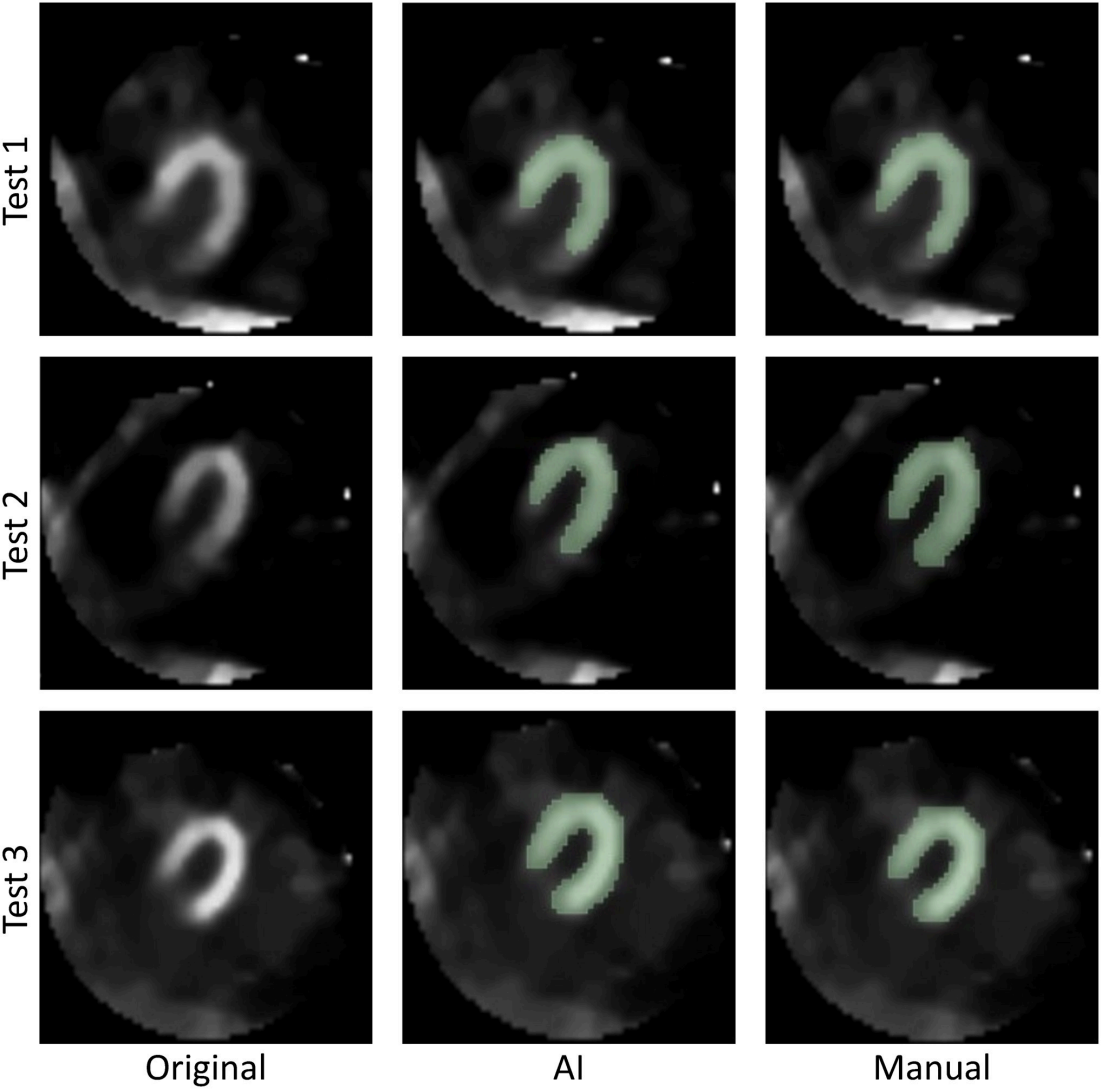
Images chosen at random and forwarded to the *M*_*N*_ in charge of evaluation.

Three nuclear medicine specialists, $M_{N_{1}}$ with 18 years of experience, $M_{N_{2}}$ with 20 years of experience, and $M_{N_{3}}$ with 12 years of experience, independently and blindly evaluated the delineation of the left ventricle walls in each mask. One mask was manually created and used for training the network, and the other was generated by the proposed AI model, both focusing exclusively on the left ventricle walls. This analysis showed a high degree of agreement among the specialists most of the time, with evaluations indicating between 80% and 100% concordance in the delineation of the masks in both groups, as shown in Table 1. Fig 3, extracted from Nogueira *et al.* [73], visually presents the results, displaying Test 1, Test 2, and Test 3 in the rows, and the original images, the masks generated by AI, and the masks manually created by the specialists in the columns, respectively.

**Table 1:** General medical evaluation of the tests.

Scale items	AI	Manual
I agree 100% with the mask	4	4
I agree 80% with the mask	5	4
I agree 60% with the mask	0	1

Table 2 presents the individual assessments of the tests and their agreement indices. In the qualitative analysis conducted by the medical specialists *M*_*N*_, at least 80% agreement was observed regarding the positioning of the masks generated by the proposed model. In Test 1, which recorded the greatest divergence among the evaluators, the lowest score was assigned to the manual mask. The discrepancy was primarily due to the proximity of the heart to the artifact zone, resulting in 60% agreement in the positioning of the manual mask.

**Table 2:** Individual medical assessment by test.

	Evaluation					
	Test 1		Test 2		Test 3	
Scale items	AI	Manual	AI	Manual	AI	Manual
I agree 100% with the mask	1	0	1	2	2	2
I agree 80% with the mask	2	2	2	1	1	1
I agree 60% with the mask	0	1	0	0	0	0

This study demonstrated the efficiency of AI techniques in identifying the left ventricular walls in MPS images, necessary for the diagnosis and treatment of coronary artery disease (CAD). The AI model achieved a Dice coefficient of 87% and an average Intersection over Union (IoU) of 0.8, aligning with the manual segmentations performed by specialists. The objectives were met, highlighting the model’s accuracy and feasibility in medical practice, significantly advancing medical imaging by improving diagnostic precision and optimizing processes, establishing a solid foundation for the continued development of AI in healthcare.

### Unsupervised model for brain structure segmentation

This study presents an unsupervised method for segmenting brain computed tomography scans. The proposed methodology involves image feature extraction and the application of similarity and continuity constraints to generate segmentation maps of the anatomical head structures. Designed for real-world datasets, this approach utilizes a spatial continuity scoring function adapted to the desired number of structures. The primary objective is to assist medical specialists in diagnosis by identifying regions with specific anomalies.

The unsupervised methodology produces segmentation masks, in contrast to the results generated by the CTSeg tool. To test the AI model, the Computed Tomography Quality 500 (CQ500) database, containing 500 brain CT scans performed by various hospitals in India, was used. This dataset was included in the ISMIR PACS to test data from another platform and the AI model for volumetry. The images are high quality and properly anonymized, along with clinical reports prepared by three radiologists with 8, 12, and 20 years of experience in interpreting brain CT scans.

**Fig 4:**
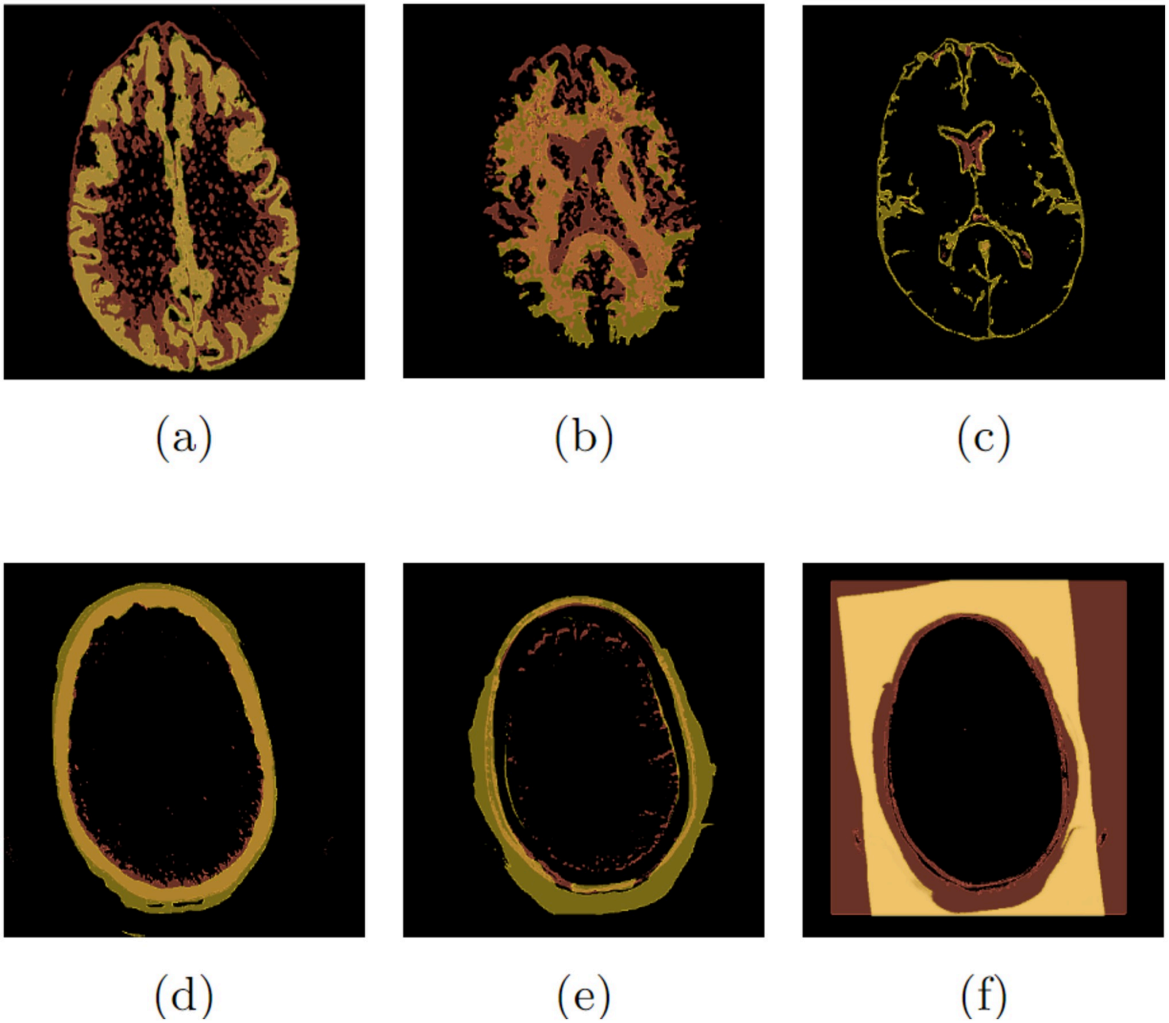
Segmentation overlap of the CT195 exam: (a) *c*_1_, (b) *c*_2_, (c) *c*_3_, (d) *c*_4_, (e) *c*_5_ e (f) *c*_6_.

Six exams were selected from the set of 500 exams. A total of twelve simulations were performed for each label or class: gray matter *c*_1_, white matter *c*_2_, cerebrospinal fluid *c*_3_, skull bone *c*_4_, soft tissue *c*_5_, and background *c*_6_. The gold standard segmentations used to calculate *D*_*c*_ are from the CTSeg tool, and the calculation of *D*_*c*_ is performed using 3DSlicer. Fig 4, extracted from Santos *et al.* [74], shows the overlap of the CT195 exam segmentation performed by the CTSeg tool and the segmentation obtained by the proposed method. By relating the six labels used by CTSeg to the labels obtained by the proposed method, it was possible to identify overlap in all classes.

For the segmentation of classes *c*_1_ and *c*_2_, shown in Fig 4(a) and 4(b) respectively, the result in the dark shade represents the entire set, while the segmentation by the CTSeg tool in the light shade is the correct subset. In contrast, for the segmentation of classes *c*_3_, *c*_4_, and *c*_5_, shown in Fig 4(c)–4(e) respectively, there is an intersection where the segmentation by the CTSeg tool in the light shade is the set and the segmentation by the proposed method in the dark shade is the correct subset. Finally, for the segmentation of class *c*_6_, shown in Fig 4(f), the CTSeg tool incorrectly considered classes *c*_4_, *c*_5_, and *c*_6_, resulting in inaccurate segmentation, while the proposed method performed the segmentation correctly.

The developed unsupervised segmentation method achieved an accuracy exceeding 65%, with notable performance in white matter segmentation and results similar or superior to CTSeg in cranial volumetry. Experts recommended a range of 3≤ labels ≤6 for the best visual results. The methodology demonstrated efficiency in training the neural network, reducing resources, training time, and costs, and facilitating the early detection of anomalies in brain CT scans.

### Pulmonary embolism classification

This study presents a classification model using AI for the detection of pulmonary embolism (PE) in computed tomographic pulmonary angiography (CTPA). The proposed model, developed from ISMIR data, utilizes a two-dimensional approach that integrates temporal series to classify each slice of the examination and make predictions at both slice and examination levels. The training process consists of two stages: first, using the InceptionResNet V2 convolutional neural network, and then, the long short-term memory (LSTM) recurrent neural network model.

Randomly, 800 exams were collected from ISMIR data, consisting of 400 positive and 400 negative for PE, with a maximum limit of 300 slices per exam. The Python pydicom library was used to load the exams, totaling 181,747 slices, with 16,492 positive and 165,255 negative, as shown in Table 3. Automatic segmentation was performed for each exam to identify the lung region. In this region, windowing was applied with a window of 1,400 and a level of -600. Completing the segmentation, background removal operations, edge region extraction, and morphological operations of erosion, dilation, OTSU thresholding, and closing were performed using the Python OpenCV library to generate the mask applied to the image. The original resolution of each slice was maintained at 512 × 512 pixels. To highlight the region of interest for PE classification, windowing with a level = 100 and width = 700 was applied to each slice. Each image was converted from DICOM to PNG 8-bit.

**Table 3:** Dataset information.

Original from ISMIR	Amount of data
CTPA (stacks)	800
Positive	400
Negative	400
CTPA (slices)	181.747
Positive	16.492
Negative	165.255

**Fig 5:**
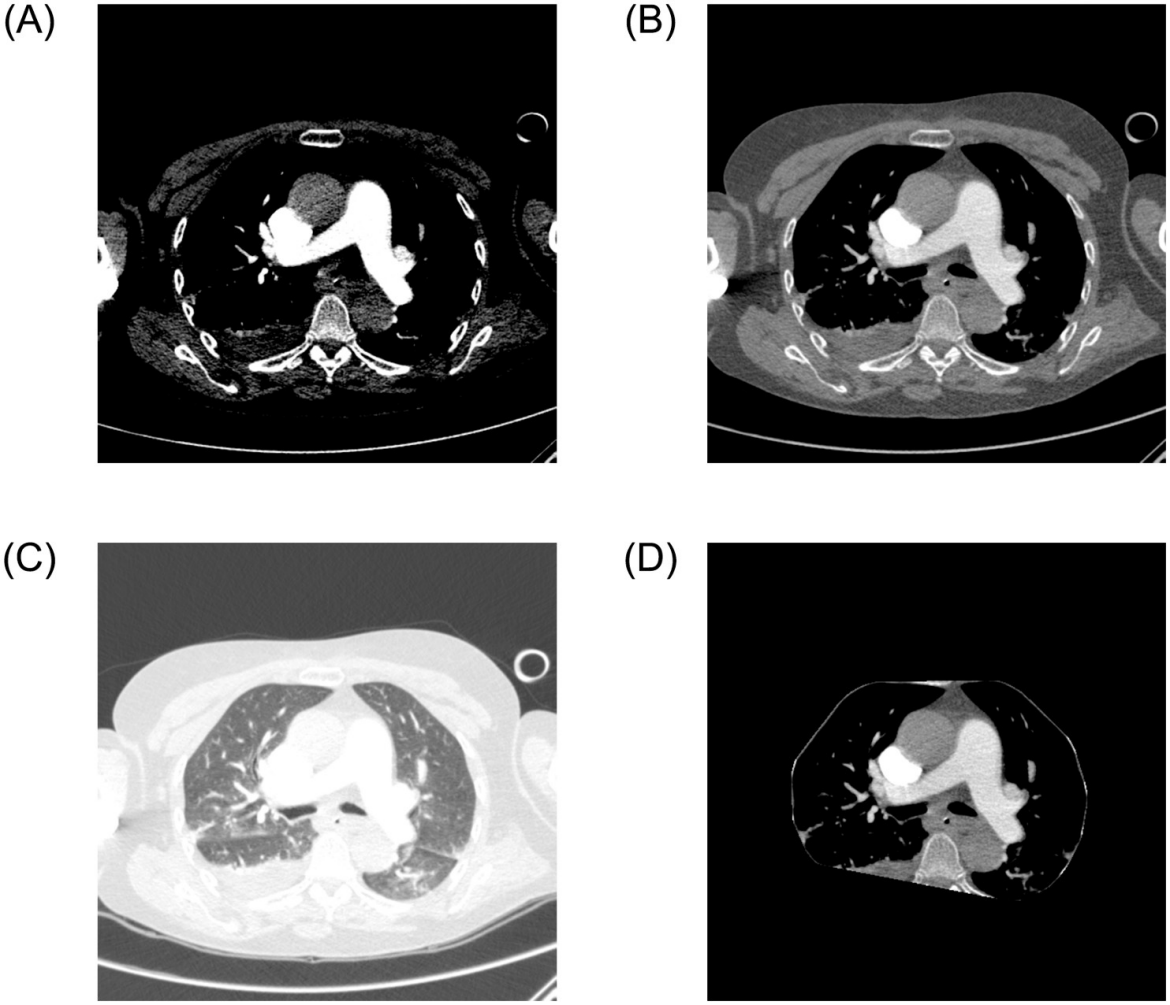
Application of automatic windowing and segmentation: (a) original image, (b) windowing for PE, (c) windowing for lungs, and (d) automatic segmentation.

Subsequently, data augmentation was performed, generating new images to improve the generalization of the InceptionResNet V2 CNN model by applying rotation, translation, brightness alteration, among others. Ten variations were created for each positive slice, totaling 164,920 images. The number of variations in data augmentation was determined by dividing the original number of negative slices by the number of positive slices. InceptionResNet V2 was trained to learn the features of positive or negative slices for PE. Only images with the visible lung region were considered valid, resulting in the exclusion of 50 positive slices. A total of 330,125 slices were considered for training the 2D model, with 164,870 positive and 165,255 negative. The feature encodings of each exam were extracted from the penultimate layer, the dense layer. Finally, the RNN LSTM model was trained to generate predictions at the image and exam levels. Both techniques, CNN InceptionResNet V2 and RNN LSTM, were coded using the Keras/Tensorflow libraries. Fig 5, extracted from Silva *et al.* [75], shows the application of windowing and the proposed automatic segmentation.

Therefore, the model adopting the CRNN classification approach was implemented, and the 2D and 2D+LSTM models were trained using cross-validation with *k* = 5 for 25 and 50 epochs, respectively. The NVIDIA TeslaT4 16GB was used to train the model. The hyperparameters of the InceptionResNet V2 model were configured as follows: i) sigmoid activation function, ii) binary cross-entropy loss (BCE), iii) Adam optimizer, iv) learning rate *η*_*adam*_ = 0.0005, and v) batch size *T*_*L*_ = 16. The hyperparameters of the LSTM model were defined as follows: i) sigmoid activation function, ii) binary cross-entropy loss (BCE), iii) Nadam optimizer, iv) learning rate *η*_*nadam*_ = 0.005, and v) batch size *T*_*L*_ = 16.

The study developed a deep neural network to classify PE in angiography images, achieving 93% accuracy at the slice level and 77% at the exam level. Validation on hospital data showed 86% accuracy for positive PE cases and 69% for negative cases. The model excelled in excluding PE, with 73% precision and 82% recall, demonstrating its clinical applicability. The demographic diversity of the validation set reinforced the model’s generalizability, indicating its potential to improve diagnosis and patient outcomes.

### Performance and security metrics protocol for ISMIR

Performance metrics are utilized at various stages of the ISMIR workflow, including: i) system availability, which measures the time the system is operational, ii) response time, which assesses the speed of response to requests, iii) storage and management capacity for large volumes of medical images, iv) data transfer rate, which measures the speed of data transfer, v) efficiency in image retrieval, vi) security and compliance with regulations such as HIPAA and GDPR, and vii) user satisfaction. Other metrics include the number of exams annotated by radiologists with and without AI and the maximum number of exams processed by AI per day.

Performance analyses were conducted for system integration using three types of exams: tuberculosis, melanoma, and neurological, with 1,690 exams of each type. Among the tuberculosis exams, 426 were analyzed by AI models, for melanoma, 141, and for neurological exams, 143. The remaining exams were returned for manual processing or data correction, as illustrated in the flow of Fig 1. Common errors detected include corrupted FHIR arriving at the dispatcher, lack of AI for FHIR requiring manual processing, presence of AI and FHIR but with model errors, AI inference failures, and model persistence failures of AI inferences. These issues were addressed by dividing the system flow into two parts: i) the main flow and ii) the alternative or exception flow.

The main flow is followed when all data is correct, the alternative flow occurs when there is no AI model for the exam, and the exception flow is used for unexpected subsystem failures. Errors and failures are restored in real-time without system downtime. Performance analyses used the same exams: tuberculosis, melanoma, and neurological, with 1,690 exams each. The average data transfer times are 20*s* for tuberculosis, 59*s* for melanoma, and 2*m*05*s* for neurological. Tuberculosis exams are single black-and-white images, melanoma exams are single color images, and neurological exams are three-dimensional black-and-white images.

Regarding data storage in ISMIR, the expected load is 16,000 exams per day. In the worst-case scenario, considering volumetric neurological DICOM exams of approximately 1GB each, this equates to 16TB per day for SUS. This was the test conducted for data storage statistics. For expected data input growth, ISMIR was stress-tested by inserting approximately 40,000 exams in a single day without failures. Security measures for confidential data transmission include: encryption in transit (SSL/TLS), encryption at rest in VNA, MongoDB, and S3/MinIO, multifactor authentication, role-based access control (RBAC), audit logs, continuous monitoring, secure access tokens, rate limiting, network segmentation, firewalls, updates and patches, secure configurations, sandboxing, and strict anonymization process control policies.

To avoid performance errors in diagnosis, extensive validation tests were conducted using a comprehensive dataset to ensure the system’s proper functioning. Cross-validation was performed by comparing diagnoses produced by radiologists with the system’s automatic segmentations. Training, retraining, and updating of models are carried out periodically with new data and techniques to improve segmentation accuracy. Quality monitoring occurs in real time to identify error patterns and notify administrators of significant deviations. In critical cases or when uncertainty arises, manual segmentation review is established. Double reading, where multiple radiologists review automatic segmentations, is performed to avoid biases.

Specific tests are conducted, including system availability and reliability tests to verify the Gateway and VNA uptime. Response time and latency tests are applied to the anonymizer, standardizer, VNA response, MongoDB and S3/MinIO, dispatcher, and DicomWeb. Network performance and data transfer tests evaluate transfer rates and FHIR compliance. Processing and modeling tests measure model management efficiency, AI model execution, accuracy, and adequacy. Display and result tests verify image display and predictive results. Failure and recovery tests include recovery mechanisms and failure notifications. Integration tests ensure compliance with international medical integration protocols.

Regarding the impact of the system on healthcare cost reduction, several tuberculosis exams were analyzed by AI and radiologists, including conditions such as atelectasis, opacity, edema, pleural effusion, mediastinal widening, cardiomegaly, pneumothorax, consolidation, and lung lesions. Kazemzadeh *et al.* [76] demonstrated that AI identification of tuberculosis for NAAT confirmation reduced specialist time by 40% to 80% per patient. Viney *et al.* [77] reported that a two-view chest X-ray at the Centers for Disease Control and Prevention, Atlanta, costs approximately $34. The Brazilian labeled chest X-ray dataset (BRAX) [69] contains 24,959 chest X-ray studies, totaling 40,967 images verified and anonymized by trained radiologists. Without AI, costs would be $848,606. With AI segmenting the exams and subsequent radiologist analysis, there is an average reduction of 60%, totaling $339,442, saving approximately $509,163. These values are modest, considered below the market average.

The need to develop AI models is based on the priority queue of SUS. This queue prioritizes people with physical disabilities, seniors aged 65 or older, pregnant women, lactating women, and individuals with young children, as mandated by law. In the PACS, as the number of exams increases, bottlenecks such as the need for reports, exam analysis, and annotations arise. Therefore, models are created according to SUS priorities and these bottlenecks. The protocol ensures that AI models initially present positive results for diseases and are then refined for segmentations and other tasks. Once the AI model is built, tested, and validated, it is put into production. Periodically, AI models are retrained with new patient data for better generalization. Currently, this retraining process occurs manually since most models are supervised and require targets.

## Discussion

The three case analyses demonstrated significant benefits for healthcare. In the first analysis, the AI model improved the identification of left ventricular walls in myocardial perfusion scintigraphy images, aiding in the diagnosis and treatment of coronary artery disease. The second analysis presented an efficient unsupervised method for brain CT segmentation, facilitating precise diagnoses of structural anomalies. The third analysis, involving the classification of pulmonary embolism in CT angiographies, showed high accuracy in detecting and excluding PE, optimizing the diagnostic process. These analyses are detailed in Nogueira *et al.* [73], Santos *et al.* [74], and Silva *et al.* [75]. After implementing the AI models requested by the Brazilian Ministry of Health, a new study will begin to include new exams in the training of existing models, updating them with new data.

In the deployment of ISMIR, various tests have been conducted to assess operational performance. These include maximum load and transfer tests to verify image reception capacity, storage and data retrieval capacity tests to ensure sufficient space and efficiency in recovery, response time tests, including latency and processing of AI models, as well as integration tests to ensure cohesive operation between systems. Data security tests and compliance with regulatory standards are also performed. These evaluations are needed to ensure the effectiveness, efficiency, reliability, and security of the systems in healthcare environments. After these tests, the analyses demonstrate the capability to receive thousands of exams daily, with AI models processing hundreds of exams per day, resulting in a system in production, active, operating as planned, and ready for continuous use in the clinical setting.

The successful deployment of ISMIR covered aspects such as system configuration, data network, storage, data compression, image input, and display, communication, and data security, providing significant benefits to the IT team, specialized doctors, patients, and ISMIR as a whole. The IT team at ISMIR gains broad and specific benefits, including operational efficiency, data integration, automatic reporting, information security, technical support, technological updates, and emergent management, as well as data security and recovery. Specifically, the assessment of system efficiency in medical image communication, monitoring the performance of PACS, VNA, and AI models, ensuring interoperability between healthcare systems, secure data sharing, report automation, deployment of standards and security measures, continuous evaluation of practices and regulatory compliance, monitoring system integrity, and efficient technical support stand out. By focusing on these outcomes, the IT team contributes to the overall efficiency of the system, ensuring that technological solutions meet the operational and security needs of ISMIR.

For ISMIR-specialized doctors, the benefits encompass improved diagnostic accuracy, automated reporting, data integration, and sharing, as well as patient history. More specific aspects include assistance in detecting complex patterns in medical images, increased accuracy in diagnostic interpretations, identification of imperceptible anomalies to the naked eye, support in image analysis for personalized treatment plans, contribution to identifying patient-specific characteristics, standardization and consistency in reports, improvement in interoperability between healthcare systems, ease in secure sharing of images and information, storage of medical images, and facilitation of the review and comparison of previous exams. These outcomes provide advantages for healthcare professionals, resulting in more precise diagnoses, better treatment coordination, and overall enhancement in ISMIR efficiency.

For the patient, the benefits that most directly impact their experience in ISMIR include an increase in diagnostic accuracy, more reliable information about their health condition, a higher likelihood of receiving a personalized treatment plan based on detailed analyses of their medical images, efficiency in the communication of exam results, ease in sharing their health information with different professionals and institutions, resulting in a complete medical history, access to the medical history over time allowing a more comprehensive view of their health, and increased confidence in the security and confidentiality of their medical information. These outcomes present a more patient-centered experience, where diagnostic accuracy, treatment personalization, and efficient access to medical information contribute to individual health management.

ISMIR experiences benefits that reflect management efficiency, operational improvement, and efficient data integration among systems. Technological advancements facilitate quick and accurate interpretations of exams, enabling personalized treatments based on detailed analyses. The automatic generation of standardized reports and optimization of healthcare professionals’ workflow contribute to an efficient, patient-centered approach. Furthermore, information security practices strengthen patient preference for ISMIR, offering a comprehensive history of exams and information. These combined results propel ISMIR towards advanced practices, promoting high-quality services, proactive community health management, and financial cost reduction.

One of the main challenges encountered was the integration of the DICOM standard into ISMIR, requiring deep knowledge of medical protocols, digital imaging expertise, and device interfaces. ISMIR provides an API for DICOM integration into healthcare applications and devices [78]. Choosing DICOM required careful resource analysis, software configuration, and technical support. AI models for medical image segmentation offer various aids to radiologists, including rapid anomaly identification, workload reduction, diagnostic standardization, structure quantification, hidden pathology detection, clinical decision support, disease monitoring, hospital system integration, and support for training and education. These tools enhance diagnostic time, operational efficiency, and patient care quality, providing detailed disease progression tracking. Radiologists are pivotal in this process, as manual segmentations serve as the gold standard for both supervised and unsupervised processes. Another important task for radiologists is the validation of all AI segmentation models.

With a focus on cost reduction, an analysis can be conducted to assess the savings achievable by reducing the number of hours worked by radiology specialists. Ramalho *et al.* [79] work delineates the continuous expansion and refinement in the field of radiology, which has experienced significant growth in demand in recent years, currently ranking as one of the most highly regarded and advantageous medical specialties. Financial gains in this field are among the highest reported, such as US$ 400,000 in Canada, US$ 375,000 in the USA, US$ 317,000 in Austria, US$ 200,000 in the Netherlands, US$ 183,000 in Germany, and US$ 120,000 in Denmark. When comparing the number of radiology specialists per 1000 inhabitants, Brazil has 0.0389, and Turkey has 0.0322, both with a low number of radiology specialists compared to countries like Denmark with 0.1857, Austria with 0.1073, and the USA with 0.0874.

When a radiology specialist needs to perform manual segmentations and annotations on medical imaging exams, each slice of the exam is meticulously analyzed. Typically, exams start with 50 slices, consuming a significant amount of time. However, using AI models for segmentation reduces the radiologist’s workload by approximately 60% [76], resulting in substantial cost savings in a single medical specialty. Regarding AI models, the study highlights strengths in high validation accuracy, with near 98% agreement, and practical application, meeting the country’s needs. Nevertheless, limitations include variability in image quality due to diverse devices, dependence on manually annotated data, high initial implementation costs, continuous retraining needs, and variability in acquisition protocols, which can impact segmentation efficiency.

In terms of ISMIR implementation, strengths include the replacement of radiographic films with digital images via PACS, allowing quick and secure access, and solving system incompatibilities with VNA. The platform ensures resilient security with encryption and compliance with international standards. Limitations involve challenges in integrating the DICOM standard in some devices, maintenance complexity to ensure data integrity and availability in distributed storage platforms like MongoDB or HDFS, requiring continuous and complex management, and high initial implementation costs, despite long-term savings projections.

The diversity of imaging diagnostic devices can affect exam quality and segmentation efficiency. AI should be trained with representative data from various sources for proper generalization. AI performance can vary due to acquisition quality and consistency among machines and imaging protocols, including variations in acquisition, resolution, noise levels, and calibration. AI, especially deep learning algorithms, is sensitive to image quality. Training AI with diversified datasets and using normalization and preprocessing techniques can mitigate these differences. Zhang *et al.* [80] describe that machine diversity can make models more resilient and promote their widespread use. A practical example is the second study in this work, which used the CQ500 dataset, consisting of exams from multiple hospitals in India with different devices. In this case, diversity was beneficial, introducing variability in the proposed methodology and enabling better evaluations with different CT scanner configurations.

## Conclusion

This work, the result of collaboration among various institutions, researchers, and expert professionals, aims to deploy the Integrated System for Medical Image Repository (ISMIR) platform in an extensive public healthcare system. The primary objective was achieved in the year 2023, with the successful proof of concept, which was tested and validated. The project’s primary hypothesis was confirmed, as the system accelerated the deployed processes. Following the system’s deployment, various other computational intelligence modalities can be incorporated, including the action that utilizes the Manchester Triage System (MTS) to identify exam priorities, prioritizing patients with greater urgency. Therefore, it is concluded that ISMIR can connect to the entire country, receiving thousands of images daily and conducting hundreds of assessments through the deployed AI models. Moreover, it maintains the ability to continue implementing AI models and intelligence to expedite the analysis process in medical image exams.

## Supporting information

S1 File (DOC)
