# Supplementary material for: Pilot deployment of a cloud-based universal medical image repository in a large public health system: A protocol study
Source: PLoS One. 2024 Aug 29;19(8):e0307022. doi: 10.1371/journal.pone.0307022 (PMC11361589; doi:10.1371/journal.pone.0307022)
Supplement: S1 File — (DOC) [file pone.0307022.s001.doc]

**Supporting information**

**PI – Project information:** The PROADI Image Bank (BCIM) was initiated on January 1, 2019, under the title **Expansion of the National Platform for Storage and Artificial Intelligence in Medical Images for Research, Innovation, and Clinical Decision Support**. Its objective is to develop the National Platform for medical images within SUS/Brazil, fostering collaboration between hospitals and AI developers. In the initial phase, BCIM-I, the PACS was implemented with the VNA for secure image storage. A proof of concept for AI-based diagnostic analyses was developedfor Melanoma, Tuberculosis, and Congenital Zika Virus Syndrome. The project was divided into four subprojects, each obtaining the Certificate of Presentation for Ethical Appreciation (CAAE) with the following numbers: CAAE 32903120.4.0000.0071 for Melanoma, CAAE 52257521.8.0000.0071 for Neurological, CAAE 52241121.0.0000.0071 for Tuberculosis, and CAAE 74792423.5.0000.0071 for the Universal Platform. BCIM involves 49 researchers, 30 institutions, and 30 scholarship recipients. For more details, please visit the link (in Portuguese): https://bancodeimagens.io/.
